# AC/DC Current Sensor for Rotating Applications

**DOI:** 10.3390/s20236811

**Published:** 2020-11-28

**Authors:** Miguel Angel Pardo-Vicente, Carlos A. Platero, José Ángel Sánchez-Fernández, Francisco Blázquez

**Affiliations:** 1 Department of Hydraulic, Energy and Environmental Engineering, Universidad Politécnica de Madrid, 28040 Madrid, Spain; ma.pardo@alumnos.upm.es (M.A.P.-V.); joseangel.sanchez@upm.es (J.Á.S.-F.); 2 Department of Automática, Ingeniería Eléctrica y Electrónica e Informática Industrial, Universidad Politécnica de Madrid, 28006 Madrid, Spain; francisco.blazquez@upm.es

**Keywords:** alternate current, direct current, sensor

## Abstract

There are several techniques for current measurement. Most of them are capable of measuring both alternating and direct current (AC/DC) components. However, they have severe drawbacks for rotating applications (large size, sensitivity to external fields, and low signal amplitude). In addition to these weaknesses, measured signals should be transmitted to a stationary part. In order to contribute solving these difficulties, this paper presents a sensor that can measure AC/DC simultaneously based on the electromagnetic coupling of two coils. To this aim, the measured waveform is analysed. In this paper, the design of such a sensor is presented. This design is validated through computer simulations and a prototype is built. The performance of this sensor prototype is analysed through experimental tests.

## 1. Introduction

The value of electrical current is a critical parameter in many processes. For this reason, current measurement has been made since the beginning of human-made electric current generation. Current measurement techniques can be classified according to the physical principle in what they are based [1]:

- Ohm’s law;
- Faraday’s law;
- Magnetic field sensors;
- Faraday effect.

Ohm’s-based sensors are the simplest and can sense both direct and alternating current (DC and AC) [2]. For this reason, they have been used since the beginning of electricity in the form of shunt resistors. They are cheap and robust, but for large currents, they are bulky because of their need to dissipate their inherent power losses as heat [3]. Their accuracy can be enhanced through compensation [4]. However, they are galvanically connected to the circuit where the current is being measured. This may make them impractical for some applications.

On the contrary, Faraday’s law-based sensors are inherently galvanically isolated from the circuit whose current will be measured. This characteristic allows safely measuring currents in high voltage systems. However, they are inherently ill suited to measure DC currents [5]. There are two sensor types that use Faraday’s law for current measurement: Rogowski coils and current transformers.

Rogowski coils have an air core. For this reason, they are well suited to measure high frequency currents [6]. In addition, they are also used to measure high currents [7]. However, for power industry frequency (50–60 Hz), current transformers are the most used current sensor [8]. They are at the base of relay protection of power systems [9]. Current transformer accuracy is limited by the saturation of their core [10]. Therefore, there are several approaches to detect [11] or correct [12] core saturation in measurement accuracy.

Magnetic field sensors are galvanically isolated from the main circuit. They are based on the Hall Effect, the fluxgate principle or the magneto resistance effect [1]. Hall Effect current sensors are able to measure DC and AC currents [13]. They are heavily affected by electromagnetic interference [14]. Therefore, to achieve good accuracy magnetic shielding is needed [15]. Although sensors based on the fluxgate principle can measure DC and AC currents [16], they are mainly used for DC measurements [17]. There are several magneto resistance effects that can be used to measure currents. From them, anisotropic magneto resistance (AMR) bandwidth can be extended to the MHz range [18]. Giant magneto resistive (GMR) based sensors are very susceptible to stray magnetic fields. Therefore, they should be shielded to avoid measurement errors [19]. Another magnetic effects that are used for current measurements are Giant Magneto Impedance (GMI) Effect [20] and Tunnel Magneto resistive (TMR) Effect [21].

Faraday effect current sensors are based in the phase change caused by an electric current on polarized light [22]. For this reason, they are called optical current sensors (OCS). As these procedures make light measurements, they are very well suited for current measurement in high voltage systems. In addition, they are more appropriate for high currents because their absence of magnetic saturation. Furthermore, they have a size smaller than other alternatives and provide a high bandwidth [23].

Moreover, there are current sensors based on force or strain measurements [24]. They can achieve high range but are very sensitive to external perturbations such as external magnetic fields, temperature, vibrations or gravity.

It seems that any possible application has an appropriate current sensor. However, measuring currents flowing through brushless synchronous generators rotor windings is an unsolved problem. In fact, the authors do not know any sensor that allows measuring this current.

The electrical machine most used to convert rotating mechanical energy in electrical energy is the synchronous machine, normally used as generator [25]. The synchronous generator (SG) usually has its field winding in its rotor and its armature winding in its stator. Therefore, a direct current (DC) should feed its rotating field winding. Nowadays, there are two main procedures to do this [26]:

- Static excitation system:
  This type of excitation system has no rotating component. It comprises a controlled rectifier to produce the DC excitation current for the field winding of the synchronous machine. The rectified voltage feeds, through slip rings, the field winding. The power supply of this controlled rectifier normally comes from a transformer fed from the synchronous generator terminals.
- Brushless excitation system:
  This excitation system has some rotating components, installed in the rotor of the SG. It comprises an auxiliary synchronous generator (ASG), normally known as exciter, and a rotating diodes bridge. The exciter has its field winding in its stator and its armature winding in its rotor. It is placed in the same shaft and rotates at the same speed of the main SG. The voltage that appears in the ASG armature winding is rectified by the rotating diode bridge and feeds the field winding of the main SG. As both machines rotate at the same speed, the connection between them, that feeds the rectified armature current of the ASG to the field winding of the main SG, does not have slip rings. It is brushless. The number of pair poles of the exciter is normally greater than the main synchronous machine. Therefore, its rated frequency is greater as well. This is normally done to reduce the ripple of the field current supplied.

The main advantage of the second procedure is a reduction in maintenance needs. There are no slip rings or brushes [27]. The main drawback of this procedure is the difficulty of a direct measurement of field current. Therefore, several procedures to estimate this current exist [28]. To overcome these difficulties, it is possible to install current sensors in the rotor that connect wirelessly to an external device [29]. However, this solution cannot be applied to an industrial SG where any device placed in its rotor should withstand severe centrifugal forces and vibrations. A recently proposed solution [30] is putting a Hall Effect sensor in the enclosure of the exciter close to the shaft that mechanically connects the exciter and the main SG. This proposal is oriented at measuring field currents in a superconducting brushless SG.

In this paper, a new sensor, based on the electromagnetic coupling of two coils, is presented. The first coil is placed in the rotor while the second is in the stator. The measured current flows through the first coil which induces a voltage in the second one. By the analysis of the harmonic’s components of this induced voltage, the current AC and/or DC can be measured. The sensor can be considered as a synchronous machine with a specially designed armature, at no load condition.

Although the idea behind this design is similar to that presented in [30], the proposed design is more robust. Power stations are dirty environments. Therefore, any sensor put in them should withstand vibrations, temperature changes and a noisy environment. In an industrial SG, these changes can change enough sensor distance to the shaft. This will change the sensed magnetic field and, therefore, the measurements.

This paper is organized as follows: In the Materials and Methods section the design of the sensor is presented. In addition, the simulations and the tests made to check the design are also presented. The results of both the simulations and the tests are presented in the Results section. In the Discussion section, the results of the simulations and tests are compared. Then, the strengths and weaknesses of the sensor are discussed. Finally, in the Conclusions section, some conclusions are drawn.

## 2. Materials and Methods

**Figure 1:**
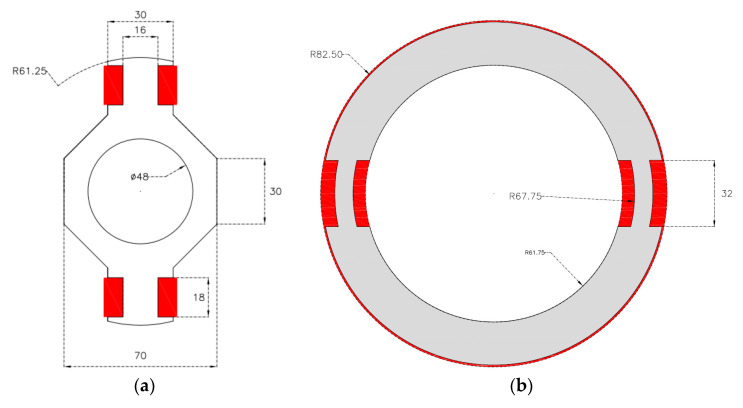
Main dimension of the rotor (**a**) and stator (**b**) of the current sensor prototype.

The proposed sensor is a synchronous machine specifically designed for measuring alternating current (AC) and/or direct current (DC). The current to be measured flows through the rotor winding. The armature winding is in the stator. However, it is placed in such way that is crossed by the yoke magnetic flux (Figure 1). It is important to remark that in a conventional synchronous machine, the armature winding is located in a different position and it is crossed by the radial magnetic flux. The electromotive force induced in the armature winding is different depending on the characteristics (AC/DC) of the field current.

When the rotor winding is fed by DC current, the machine performs like a standard synchronous machine. Therefore, the induced electromotive force will be periodic with a frequency that is proportional to the rotational speed of the machine, as it is a two-pole machine

**Figure 2:**
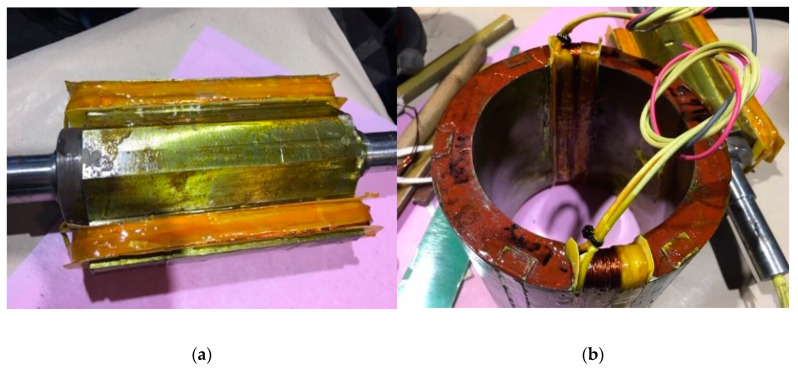
Rotor (**a**) and stator (**b**) of current sensor prototype during the manufacturing process.

When the rotor winding is fed by AC current, the Leblanc theorem determines the performance of the sensor [31]. According to this theorem, the magnetic field produced by a winding fed by AC current is equivalent to the sum of two rotating fields. One with the same sign that rotor rotation and the other rotating in the opposite direction. Therefore, the electromotive force induced in the armature windings will be the sum of two sinusoids with a frequency that corresponds to the AC that feeds the field winding plus a term proportional to rotational speed and minus this term, respectively. To test this approach, a sensor prototype has been built. Figure 1 shows a scheme of the prototype and Figure 2 shows its stator and rotor. The main characteristics of the prototype are summarized in Table 1.

The performance of this prototype has been analysed through finite element simulations. These simulations were made with two different aims. The first aim was determining the maximum currents that can be applied to the prototype without saturating its magnetic core. The second aim was to model actual operating conditions when the sensor is fed by DC, AC, or a combination of both currents.

It has also been validated by experimental tests. For this purpose, two slipping rings and two brushes have been installed. The current injection has been performed with a current injection test device.

## 3. Results

### 3.1. FEM Simulations

These simulations have been made with two different aims. On one hand to evaluate magnetic core saturation. On the other hand, to analyse sensor response to AC and DC excitation currents.

It should be considered that one of the main sources of inaccuracies in magnetic measurement systems is magnetic core saturation [32]. To avoid this, simulations were made looking for maximum field currents that provoke rotor or stator core saturation. The current source feeds the sensor rotor circuit. This current is

(1) $$I_{R}=I_{dc}+I_{ac}\cdot sen\left(2\cdot \pi \cdot f\cdot t\right)$$

where

$I_{R}$: Current flowing through rotor winding;
$I_{dc}$: Direct current;
$I_{ca}$: Alternating current;
f: frequency;
t: time.

#### 3.1.1. Simulations with DC Current

##### Sensor Saturation

Several simulations have been performed with the rotor winding fed by DC current. From the results of these simulations, it was determined that the sensor magnetic core saturation starts at 50 mA. Therefore, when feeding the rotor current according to Equation (2).

*I_R_* = 50 mA (2)

**Figure 3:**
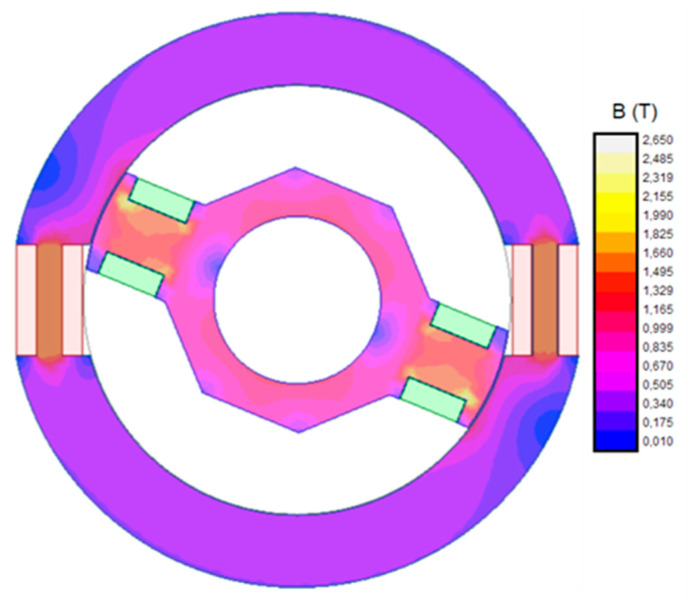
Flux density contour map of the sensor fed with 50 mA DC.

Figure 3 shows magnetic flux density in stator and rotor when this current is applied to the rotor winding and the stator circuit is open.

As shown in Figure 3, the maximum flux density is greater than 1.6 T. In both cases (stator and rotor), maximum flux density appears near the windings. In these zones, iron width is minimum. It should be emphasized that both maximums correspond with a rotor position next to horizontal axis. In this position, rotor poles pass in front of stator windings.

##### Stator-Induced Voltage

**Figure 4:**
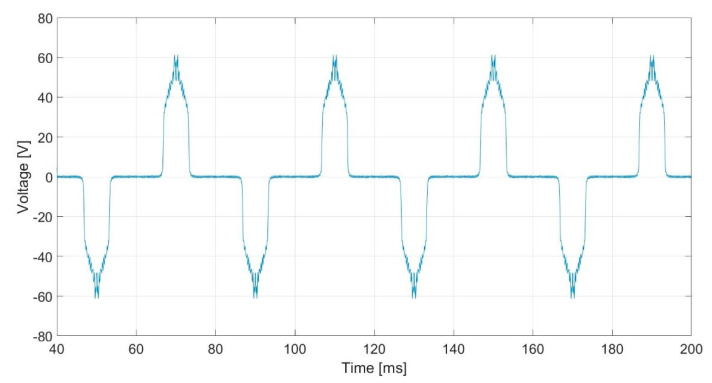
Induced voltage in case of 8.35 mA DC current in the rotor.

To analyse sensor response to DC current flowing through rotor windings, a DC current of 8.35 mA is fed to rotor windings. Figure 4 shows voltage induced in stator windings when the rotor speed is 1500 rpm. Stator voltage amplitude is maximum when the rotor poles are in front of the stator windings and null in the remaining of the stator. This voltage peak is caused by the change in magnetic flux sign crossing stator windings. The voltage wave frequency is 25 Hz, due to the rotor mechanical speed. 

**Figure 5:**
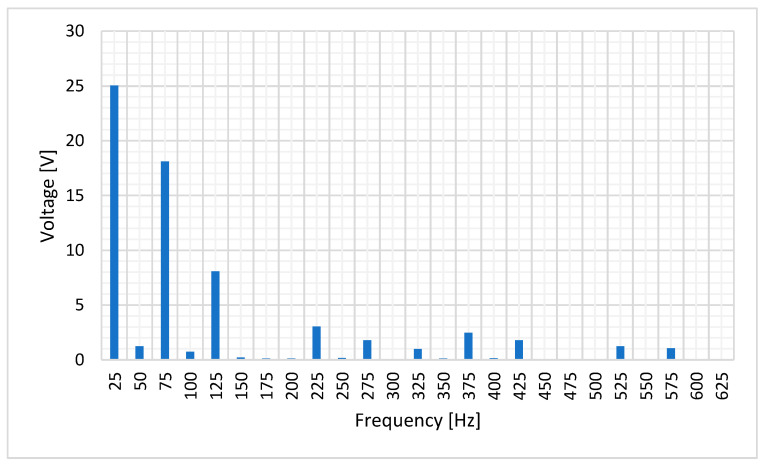
Induced voltage frequency decomposition in case of 8.35 mA DC rotor current.

Figure 5 shows the frequency components of the induced stator voltage in case of DC current injection. There are additional harmonics, but the most important component corresponds to the mechanical frequency, which is 25 Hz.

#### 3.1.2. Simulations with AC Currents

##### Sensor Saturation

When the rotor winding is fed by AC current, sensor magnetic core saturation starts from 60 mA (current amplitude) at 450 Hz. Values obtained at other frequencies are similar but those at 450 Hz are the most restrictive. Therefore, the current to be measured can be expressed as Equation (3).

(3) $$I_{R}=60\cdot sen\left(2\cdot \pi \cdot 450\cdot t\right)\;\mathrm{mA}$$

**Figure 6:**
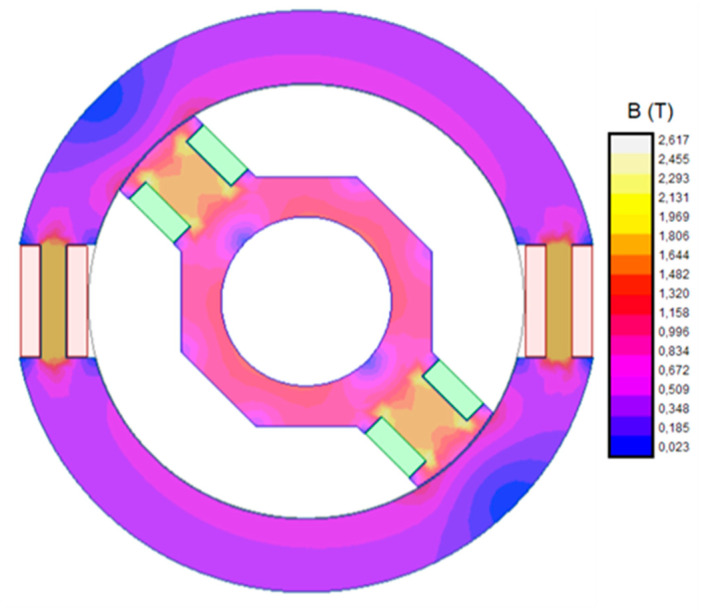
Magnetic flux density when the rotor winding is fed by 60 mA AC at 450 Hz.

Figure 6 shows magnetic flux density when this current is applied to the rotor winding and the stator circuit is open.

In this case, maximum flux density values are also near the windings. As in the DC fed case, this is due to, in these zones, the core width being at its minimum. Additionally, these maxima correspond to a rotor position near the horizontal axis because, in this position, rotor poles are crossing in front of stator windings.

##### Stator-Induced Voltage

**Figure 7:**
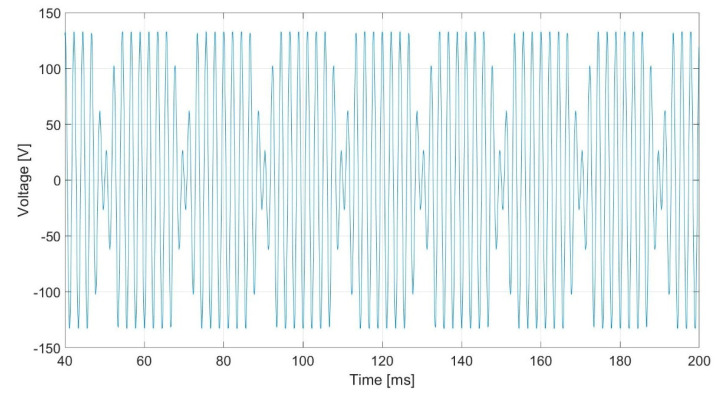
Induced voltage in case of 12 mA AC 450 Hz current in the rotor.

**Figure 8:**
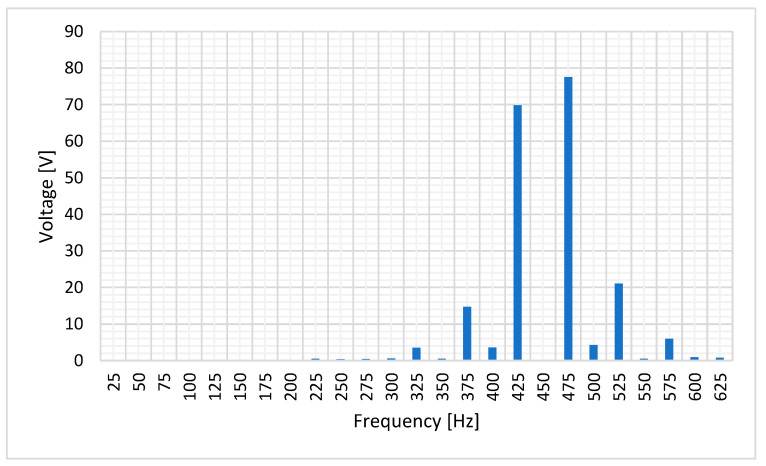
Induced voltage frequency decomposition in case of 12 mA AC 450 Hz current in the rotor.

Figure 7 shows the stator-induced voltage when the rotor speed is 1500 rpm when the current to be measured is 12 mA. Figure 8 shows the frequency components of the stator-induced voltage.

The main components of the stator-induced voltage correspond to 425 and 475 Hz. The component at 450 Hz is approximately null. As previously mentioned, this is explained by Leblanc theorem: the magnetic field has two components, one at the source frequency plus rotation speed effect (450 + 25 = 475 Hz) and the other at the source frequency minus rotation speed effect (450 − 25 = 425 Hz). As this machine has one pole pair and rotates at 1500 rpm, rotation speed effect corresponds to 25 Hz.

#### 3.1.3. Simulations with AC+DC Currents

##### Stator-Induced Voltage

The addition of 1.5 mA DC and 1.1 mA (peak value) AC (at 450 Hz) is fed into the rotor windings. Therefore, the current can be specified as Equation (4).

(4) $$I_{R}=1.5+1.1\cdot sen\left(2\cdot \pi \cdot 450\cdot t\right)\;\mathrm{mA}$$

**Figure 9:**
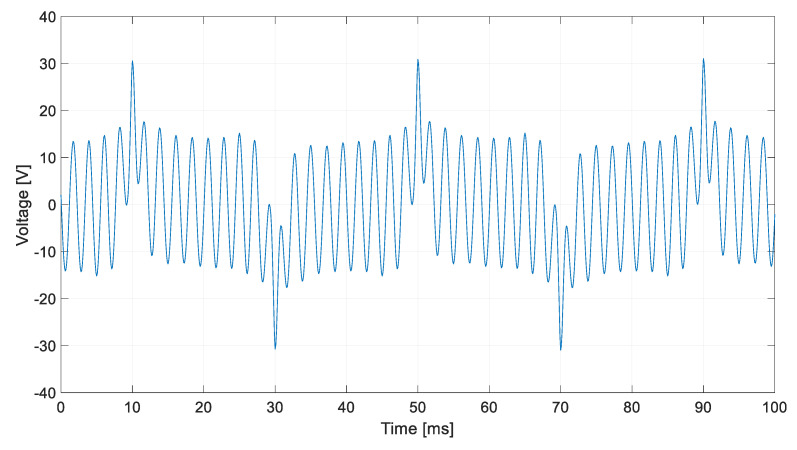
Induced voltage in case of 1.5 mA DC and 1.1 mA 450 Hz current in the rotor.

Figure 9 shows stator-induced voltage wave.

**Figure 10:**
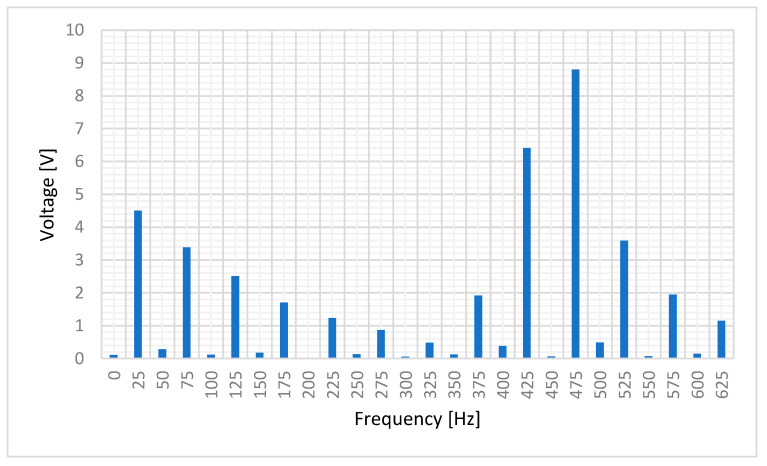
Induced voltage frequency decomposition in case of 1.5 mA DC and 1.1 mA AC 450 Hz current in the rotor.

Figure 10 shows the frequency components of the stator-induced voltage. As it can be seen in these Figures, induced voltage is the sum of the voltages that each component of the rotor current will induce in stator windings. This result agrees to the superposition principle [33].

The main frequency components correspond to 25, 425 and 475 Hz, i.e., a composition of the DC and AC cases. Therefore, the proposed sensor produces voltage waves whose characteristics depend on current characteristics. Then, processing the stator-induced voltage, the characteristics of the current that fed the rotor windings can be determined.

### 3.2. Experimental Tests

Several tests have been performed for different currents in the rotor, comprising DC, AC and DC+AC. During the tests, a programmable frequency and voltage source has been used to generate the current to be measured. This current is injected to the rotor (I_R_) coils by slipping rings and brushes. In series to the rotor coil, a 1 kΩ resistor was connected to measure the current. The induced voltage in the stator coil (V_S_) was recorded in an oscilloscope. 

These tests have been performed to corroborate that stator-induced voltage waveform and frequencies corresponds to the simulations. 

These devices have been used during the tests:

- A three-phase programmable frequency and voltage source;
- Three 4.5 V batteries;
- A variable resistor (0–5000 Ω), settled to 1 kΩ;
- A Fast Fourier Transform (FFT) two-channel oscilloscope;
- A voltmeter.

**Figure 11:**
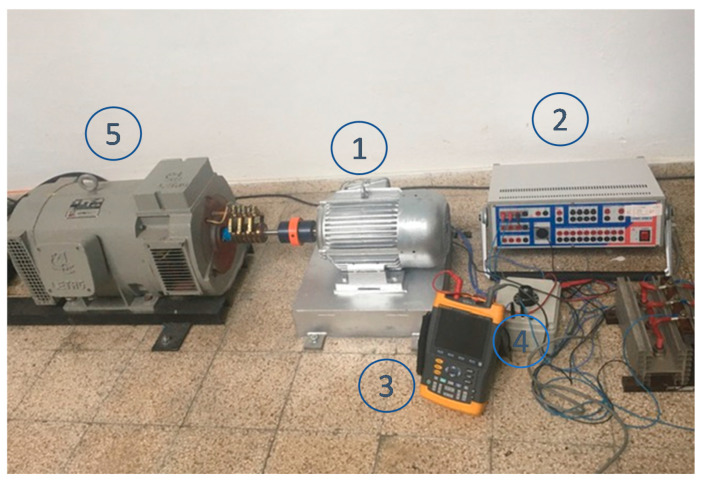
Experimental test bench, where (1) ACDC Sensor; (2) A three-phase programmable frequency and voltage source; (3) Fast Fourier Transform (FFT) two-channel oscilloscope; (4) A 5 k Ω variable resistor settled to 1 kΩ and (5) 1500 rpm synchronous motor.

**Figure 12:**
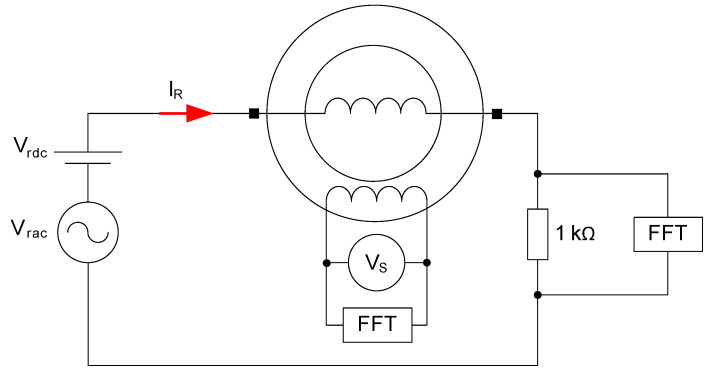
Test wiring scheme, where: (I_R_) Current in the rotor winding; (V_rdc_) DC voltage injected in the rotor test circuit; (V_rac_) AC voltage injected in the rotor test circuit; (V_S_) Induced voltage in the stator winding and FFT.

Figure 11 shows the test bench. Figure 12 shows a scheme of the measurement devices involved.

Current wave will be measured as voltage drop in the 1 kΩ resistor. The current measurements have been compared with stator-induced voltage. Motor speed was 1500 rpm in all measurements.

#### 3.2.1. DC Fed Experimental Tests

**Figure 13:**
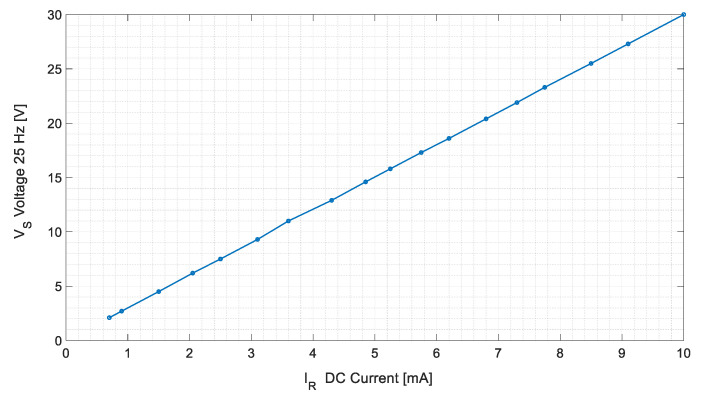
Root mean square (RMS)-induced voltage in the stator V_S_ for different DC current injection in the rotor I_R_.

Several tests, feeding the sensor with DC currents from 0.7 to 10 mA and measuring stator-induced voltage, have been made. Table 2 and Figure 13 shows the results of these tests.

**Figure 14:**
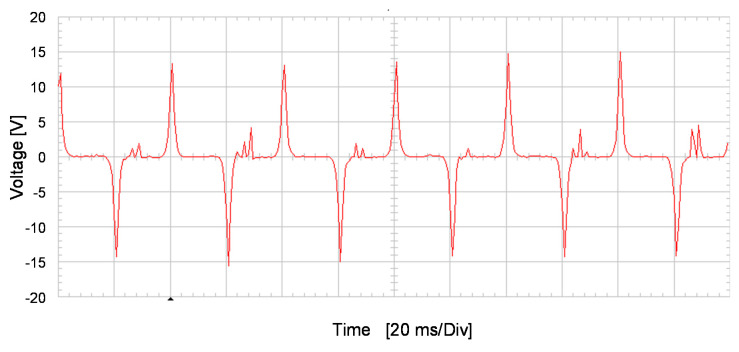
Induced voltage in the stator V_S_ for 3 mA DC current injection in the rotor I_R_.

Figure 14 shows the stator-induced voltage wave when the rotor winding is fed with 3 mA DC. In this figure an oscillating voltage which change polarity every 20 ms can be observed. This waveform is very close to that obtained in the simulations (Figure 4).

The DC current create a constant flux in the stator coil. Nevertheless, every half revolution of the rotor, the polarity of the magnetic flux changes in the stator coil. Therefore, this fact provokes a variation of the flux and, therefore a voltage is induced. In other words, the voltage is not null only when rotor poles pass in front of the stator windings

**Figure 15:**
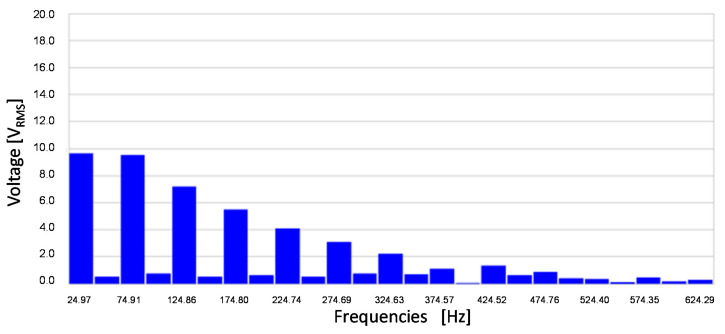
Stator-induced voltage measured by a Fast Fourier Transform Oscilloscope for 3 mA DC current injection in the rotor I_R_.

Figure 15 shows the frequency components of the stator voltage. The main components are 25 Hz and its odd harmonics. It is remarkable the 75 Hz harmonic (Third harmonic).

#### 3.2.2. Experimental Tests AC Currents

**Figure 16:**
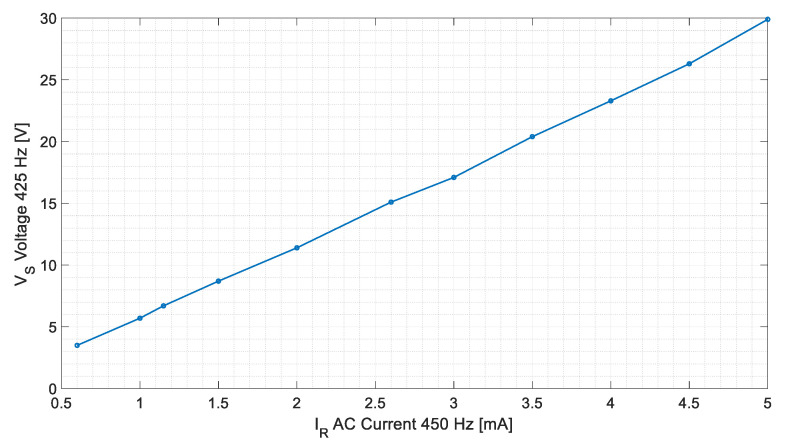
425 Hz RMS-induced voltage in the stator V_S_ for different 450 Hz AC current injection in the rotor I_R._

Several tests have been made feeding the sensor with 450 Hz AC currents from 0.5 to 5 mA, root mean square (RMS), while the stator voltages were recorded. Table 3 and Figure 16 show the results of these tests. It should be noted that the stator-induced voltages have very similar components at 425 and 475 Hz.

**Figure 17:**
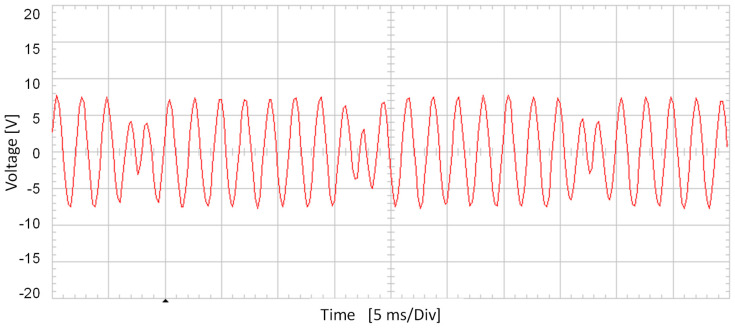
Induced voltage in the stator V_S_ for 0.8 mA 450 Hz AC current injection in the rotor.

Figure 17 shows the stator-induced voltage wave when the rotor winding is fed with 0.8 mA AC at 450 Hz. The voltage waveforms in the tests are very close to those obtained in the simulations.

**Figure 18:**
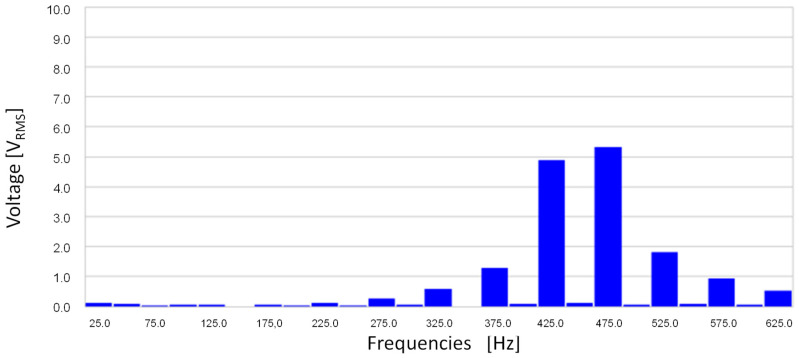
Stator-induced voltage measured by a Fast Fourier Transform Oscilloscope for 0.8 mA 450 Hz AC current injection in the rotor winding.

Figure 18 shows the frequency components of this voltage. It can be seen that the main components of stator-induced voltage have frequencies of 450 ± 25 Hz. 

#### 3.2.3. Experimental Tests DC + AC Currents

Several tests, feeding the sensor with DC currents from I_dc_ = 1 to I_dc_ = 5.15 mA plus AC currents from I_ac_ = 0.6 mA to I_ac_ = 1.5 mA RMS at 450 MHz and measuring stator-induced voltages, have been made. Three set of results are presented in Table 4a–c corresponding to DC current of ≈1.5, ≈3.0 and ≈5.0 mA for various AC currents. The voltages at 25 Hz and the voltages at 425 and 475 Hz are measured separately.

**Figure 19:**
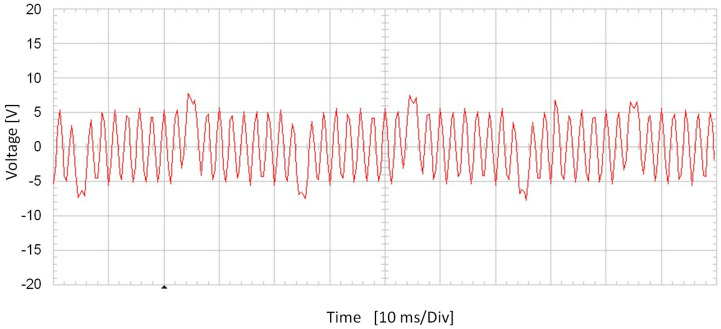
Induced voltage in case of 1 mA DC and 0.6 mA AC 450 Hz current in the rotor.

Figure 19 shows the stator-induced voltage wave when the rotor winding is fed with 1 mA DC plus 0.6 mA AC at 450 Hz.

**Figure 20:**
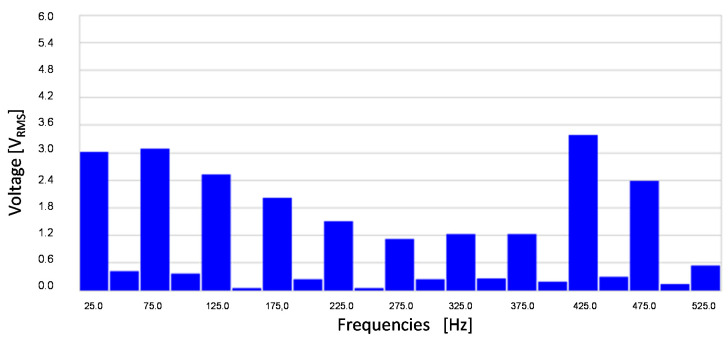
Induced voltage measured by a Fast Fourier Transform Oscilloscope for 1 mA DC and 0.6 mA AC 450 Hz current in the rotor.

Figure 20 shows the frequency components of this voltage.

It can be seen (Figure 19) the fulfilling of the superposition principle when the maximum amplitude due to the DC current couples with the effect of the AC current. Also, as in the AC tests, the main components of stator-induced voltage have a frequency of 450 ± 25 Hz.

## 4. Discussion

**Figure 21:**
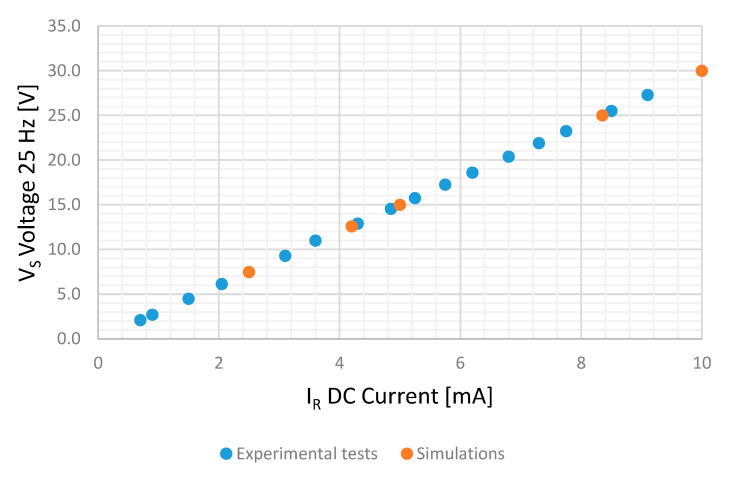
Stator-induced voltage 25 Hz component in case of DC current measurement for the experimental tests and simulations.

**Figure 22:**
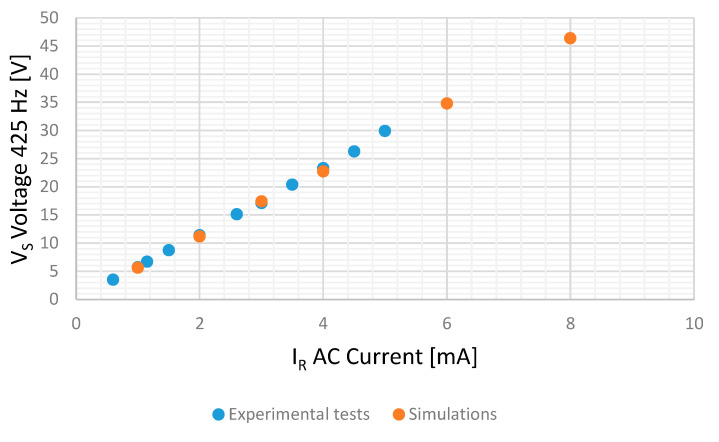
Stator-induced voltage 425 Hz component in case of 450 Hz AC current measurement for the experimental tests and simulations.

Figure 21 and Figure 22 show the experimental and simulation results in case of DC and AC measurements, respectively.

For DC tests, experimental measurements follow the line V = 2.998·I + 0.0211 with a R2 coefficient of 1.000 while simulations follow the line V = 2.9981·I − 0.0037 with a R2 Coefficient of 1.000. The difference between them is lower than the voltmeter accuracy (1.5% for a 60 V range for a frequency lower than 60 Hz).

For AC tests, experimental measurements follow the line V = 5.92561·I − 0.2594 with a R2 coefficient of 0.9992 while simulations follow the line V = 5.8391·I − 0.03425 with a R2 Coefficient of 0.9998. The difference between them is lower than the voltmeter accuracy (2.5% for a 60 V range for frequencies between 60 Hz and 20 kHz). Therefore, in all cases, the comparison between simulations and experimental tests show good agreement.

An analysis of the waveforms obtained in the stator allows determining the main components of the excitation waveform. Therefore, each component of the field current (AC and DC) can be determined. It should be noted that main components of stator-induced voltages are:

- Input signal DC → Output signal AC at 25 Hz (due to rotor speed);
- Input signal AC → Output signal AC with main components at input signal frequency ±25 Hz;
- Input signal AC+DC → Output signal is the addition of the two previous cases. It has components at 25 Hz (and its odd harmonics) and main components at input signal AC frequency ±25 Hz.

Consequently, the sensor provides, in its stator, a signal proportional to the input signal in its rotor. Therefore, it is possible to measure the input signal through an analysis of the output signal. This analysis involves the identification of voltage components of 25 Hz, which requires no less than 0.1 s. This limitation should be considered when using it.

As the correlations made to make the comparisons show, the accuracy of this sensor is higher or equal than the accuracy of the voltmeter used. Therefore, we cannot provide an accuracy for this sensor. This prototype has been tested at 1500 rpm, but due to the principles in what is based, it can be used for the same rpm range than a conventional synchronous machine.

## 5. Conclusions

Current measurement in rotating machines is not an easy task due to the centrifugal forces and severe electromagnetic environment. As field windings of brushless type synchronous machines are not accessible, measurement of their field currents is a clear example of this difficult task. 

This paper presents a current sensor for rotating machines. This sensor is able to measure both, direct and alternating currents. It is also possible, analysing sensor output, to measure any combination of AC and DC.

The current sensor comprises two coils placed in its rotor and stator. The current to be measured circulates through the rotor coil while a voltage is induced in the stator coil. The stator coil has a special design. The magnetic flux of the yoke of the stator goes through the stator winding. 

The operating principle is based on the analysis of the different components of the stator-induced voltage. Thanks to harmonics analysis, obtaining the values of the DC and AC components of the rotating current is possible.

Sensor design was evaluated by finite element simulations. In them, performance predictions were made. Then, a prototype of the sensor has been built. Finally, several experimental tests were made. In these tests, sensor performance when fed by several DC, AC and combinations of DC and AC currents were analysed.

Test results corroborate the performance of the sensor. Therefore, it is a solution for measuring currents flowing through rotating windings. These currents can be composed of any combination of AC or DC.

## Figures and Tables

**Table 1:** Current Sensor Prototype characteristics.

Variable	Value
Number of field poles	2
Number of armature poles	2
Number of turns of field winding	1000
Number of turns of armature winding	3000
Rated speed	1500 rpm
Wires section	0.096 mm^2^
Rotor diameter	122.5 mm
Air gap	0.5 mm
Machine length	170 mm
Machine weight	3.5 kg

**Table 2:** Stator-induced voltage for several rotor DC currents.

I_R_ (mA)	0.7	0.9	1.5	2.0	2.5	3.1	3.6	4.3	4.85	5.25	5.75	6.2	6.8	7.3	7.75	8.5	9.1	10
V_S_ (V)	2.1	2.7	4.5	6.2	7.5	9.3	11.0	12.9	14.6	15.8	17.3	18.6	20.4	21.9	23.3	25.5	27.3	30.0

**Table 3:** Stator-induced voltage for several rotor AC currents.

I_R_ (mA)	0.6	1.0	1.15	1.5	2.0	2.6	3.0	3.5	4.0	4.5	5.0
V_S_ (425 Hz)	3.5	5.7	6.7	8.7	11.4	15.1	17.1	20.4	23.3	26.3	29.9

**Table 4:** Stator-induced voltages for several combinations of rotor currents (DC+AC).

(**a**)												
Idc (mA)	1.25	1.30	1.35	1.50	1.50	1.50	1.50	1.50	1.50	1.50	1.50	1.50
I_ac_ (mA)	0.60	0.65	0.70	0.75	0.80	0.83	0.95	1.10	1.15	1.30	1.40	1.45
V_S_ 25 Hz (V)	3.60	3.85	4.00	4.50	4.50	4.50	4.50	4.50	4.50	4.50	4.50	4.50
V_S_ 425 Hz (V	3.49	3.75	4.10	4.40	4.70	4.85	5.30	6.40	6.70	7.60	8.20	8.40
(**b**)												
Idc (mA)	2.75	2.85	3.00	3.00	3.00	3.00	3.00	3.00	3.00	3.00	3.15	3.20
I_ac_ (mA)	0.60	0.65	0.70	0.75	0.80	0.90	1.00	1.15	1.25	1.30	1.40	1.45
V_S_ 25 Hz (V)	8.20	8.40	8.90	8.90	8.90	8.90	8.90	8.90	8.90	8.90	9.50	9.50
V_S_ 425 Hz (V)	3.50	3.70	4.00	4.37	4.66	5.10	5.70	6.70	7.10	7.58	8.30	8.60
(**c**)												
Idc (mA)	5.00	5.00	5.00	5.05	5.15	5.15	5.15		5.15	5.15	5.15	5.15
I_ac_ (mA)	0.60	0.70	0.80	0.90	1.00	1.10	1.15		1.25	1.30	1.40	1.45
V_S_ 25 Hz (V)	14.50	14.50	14.50	15.60	15.70	15.70	15.70		15.70	15.70	15.70	15.70
V_S_ 425 Hz (V)	3.50	4.08	4.68	5.12	5.75	6.60	6.70		7.15	7.60	8.40	8.65
